# Supplementary material for: Comparative Benefits and Risks Associated with Currently Authorized COVID-19 Vaccines
Source: Vaccines (Basel). 2022 Dec 1;10(12):2065. doi: 10.3390/vaccines10122065 (PMC9788423; doi:10.3390/vaccines10122065)
Supplement: Supplementary file 1 [file vaccines-10-02065-s001.zip › vaccines-1954574-supplementary.pdf]

Supplemental Figure S1 (A thru C).

A

Study or Subgroup	Vaccine Events	Vaccine Total	Control Events	Control Total	Risk Ratio M-H, Random, 95% CI
Astrazeneca	63	12082	150	11766	0.41 [0.31, 0.55]
Johnson & Johnson	195	22174	435	22151	0.45 [0.38, 0.53]
Moderna	19	15210	269	15210	0.07 [0.04, 0.11]
Pfizer	50	21720	275	21728	0.18 [0.13, 0.25]
Sputnik	79	16501	96	5476	0.27 [0.20, 0.37]

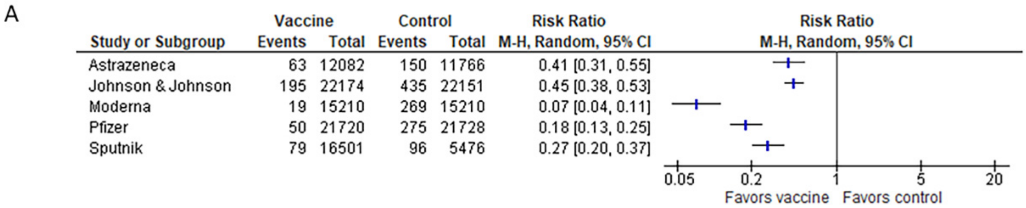

B

Study or Subgroup	Vaccine Events	Vaccine Total	Control Events	Control Total	Risk Ratio M-H, Random, 95% CI
Astrazeneca	37	12082	112	11766	0.32 [0.22, 0.47]
Johnson & Johnson	66	22174	195	22151	0.34 [0.26, 0.45]
Moderna	12	15210	204	15210	0.06 [0.03, 0.11]
Pfizer	8	21720	162	21728	0.05 [0.02, 0.10]
Sputnik	16	16501	62	5476	0.09 [0.05, 0.15]

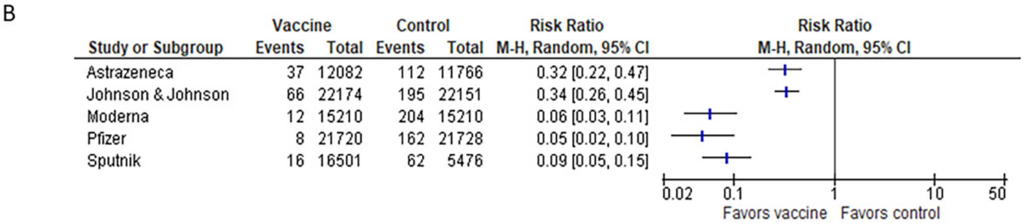

C

Study or Subgroup	Vaccine Events	Vaccine Total	Control Events	Control Total	Risk Ratio M-H, Random, 95% CI
Astrazeneca	0	12082	1	11766	0.32 [0.01, 7.97]
Johnson & Johnson	5	22174	34	22151	0.15 [0.06, 0.38]
Moderna	0	15210	30	15210	0.02 [0.00, 0.27]
Pfizer	1	21720	3	21728	0.33 [0.03, 3.21]
Sputnik	0	16501	20	5476	0.01 [0.00, 0.13]

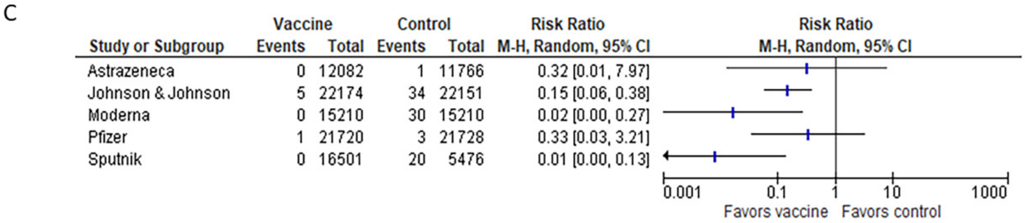

Supplemental Figure S2 (A thru B).

A.

Study or Subgroup	Vaccine Events	Vaccine Total	Control Events	Control Total	Risk Ratio M-H, Random, 95% CI
Astrazeneca	0	12082	1	11766	0.32 [0.01, 7.97]
Johnson & Johnson	0	22174	5	22151	0.09 [0.01, 1.64]
Moderna	0	15210	1	15210	0.33 [0.01, 8.18]
Pfizer	0	21720	0	21728	Not estimable
Sputnik	2	16501	0	5476	1.66 [0.08, 34.56]

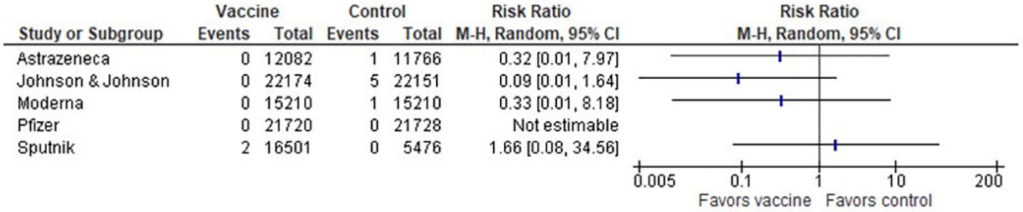

B.

Study or Subgroup	Vaccine Events	Vaccine Total	Control Events	Control Total	Risk Ratio M-H, Random, 95% CI
Astrazeneca	1	12082	4	11766	0.24 [0.03, 2.18]
Johnson & Johnson	3	22174	16	22151	0.19 [0.05, 0.64]
Moderna	2	15210	4	15210	0.50 [0.09, 2.73]
Pfizer	2	21720	4	21728	0.50 [0.09, 2.73]
Sputnik	3	16501	1	5476	1.00 [0.10, 9.57]

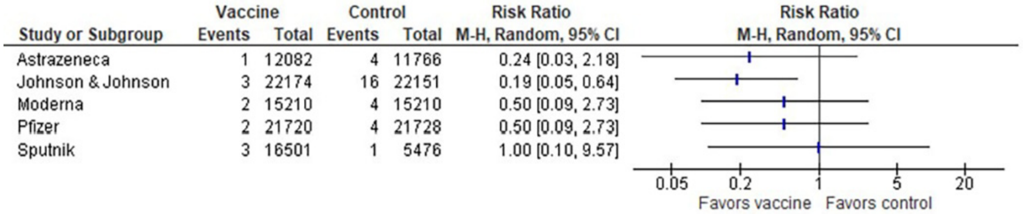

Supplemental Figure S3 (A thru B).

A.

Study or Subgroup	Vaccine Events	Vaccine Total	Control Events	Control Total	Risk Ratio M-H, Random, 95% CI
Astrazeneca	77	12021	78	11724	0.96 [0.70, 1.32]
Johnson & Johnson	83	21895	96	21888	0.86 [0.64, 1.16]
Moderna	147	15181	153	15170	0.96 [0.77, 1.20]
Pfizer	126	18860	111	18846	1.13 [0.88, 1.46]
Sputnik	43	16501	12	5476	1.19 [0.63, 2.25]

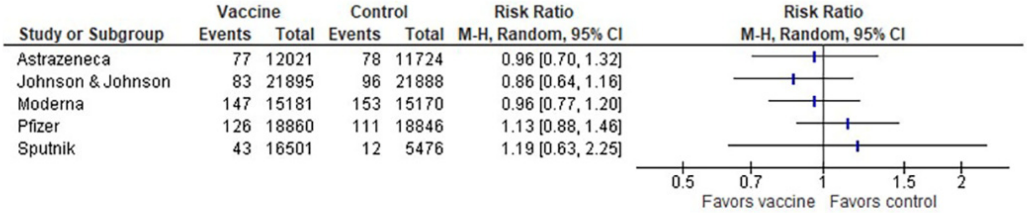

B.

Study or Subgroup	Vaccine Events	Vaccine Total	Control Events	Control Total	Risk Ratio M-H, Random, 95% CI
Astrazeneca	1	12021	1	11724	0.98 [0.06, 15.59]
Johnson & Johnson	7	21895	2	21888	3.50 [0.73, 16.84]
Moderna	6	15181	4	15170	1.50 [0.42, 5.31]
Pfizer	4	18860	0	18846	8.99 [0.48, 167.03]
Sputnik	0	16501	1	5476	0.11 [0.00, 2.72]

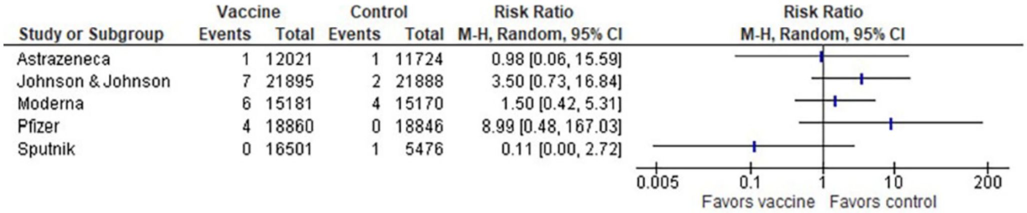

Supplemental Figure S1. Forest plot illustrating the efficacy of various vaccines for preventing A) Any symptomatic cases, B) Symptomatic cases after effective period and C) Severe cases after effective period of COVID-19 in adults. The middle line denotes the line of no-difference. The point estimate is summarized as risk ratio denoted as vertical rectangle along with 95% confidence intervals denoted as line across the vertical rectangle.

Supplemental Figure S2. Forest plot illustrating the efficacy of various vaccines for preventing A) Covid-19 related mortality, B) All-cause mortality in adults. The middle line denotes the line of no-difference. The point estimate is summarized as risk ratio denoted as vertical rectangle along with 95% confidence intervals denoted as line across the vertical rectangle.

Supplemental Figure S3. Forest plot illustrating the efficacy of various vaccines for preventing A) any serious adverse event, B) related unsolicited serious adverse events. The middle line denotes the line of no-difference. The point estimate is summarized as risk ratio denoted as vertical rectangle along with 95% confidence intervals denoted as line across the vertical rectangle.

Note: The utility of this measure is to quantify serious adverse events related to vaccination. However this information was not uniformly presented across trials, thus the most comparable set of data was utilized for this metric which may be interpreted in either of the following ways:

- Related unsolicited serious adverse events- includes all related unsolicited serious adverse events from the 3 American vaccines, as well as ALL related serious adverse events from Sputnik and AZ.
- Related serious adverse events - includes all related serious averse events except those specifically solicited in the three American vaccines.

Supplemental Table S1

Certainty assessment							Summary of findings				
Participants (studies) Follow-up	Risk of bias	Inconsistency	Indirectness	Imprecision	Publication bias	Overall certainty of evidence	Study event rates (%)		Relative effect (95% CI)	Anticipated absolute effects	
							With placebo	With Sputnik		Risk with placebo	Risk difference with Sputnik
**Any symptomatic cases**											
21977 (1 RCT)	serious[a]	not serious	not serious	not serious	none	⊕⊕⊕◯ Moderate	96/5476 (1.8%)	79/16501 (0.5%)	**RR 0.27** (0.20 to 0.37)	18 per 1,000	**13 fewer per 1,000** (from 14 fewer to 11 fewer)
**Symptomatic cases after effective period**											
21977 (1 RCT)	serious[a]	not serious	not serious	not serious	none	⊕⊕⊕◯ Moderate	62/5476 (1.1%)	16/16501 (0.1%)	**RR 0.09** (0.05 to 0.15)	11 per 1,000	**10 fewer per 1,000** (from 11 fewer to 10 fewer)
**Severe cases after effective period**											
21977 (1 RCT)	serious[a]	not serious	not serious	not serious	none	⊕⊕⊕◯ Moderate	20/5476 (0.4%)	0/16501 (0.0%)	**RR 0.01** (0.00 to 0.13)	4 per 1,000	**4 fewer per 1,000** (from 3 fewer to --)
**Covid deaths**											

Supplemental Table S1

Certainty assessment							Summary of findings				
21977 (1 RCT)	serious[b,c]	not serious	not serious	serious[d,e]	none	⊕⊕◯◯ Low	0/5476 (0.0%)	2/16501 (0.0%)	**RR 1.66** (0.08 to 34.56)	0 per 1,000	**0 fewer per 1,000** (from 0 fewer to 0 fewer)
**All-cause mortality**											
21977 (1 RCT)	serious[b,c]	not serious	not serious	serious[d,e]	none	⊕⊕◯◯ Low	1/5476 (0.0%)	3/16501 (0.0%)	**RR 1.00** (0.10 to 9.57)	0 per 1,000	**0 fewer per 1,000** (from 0 fewer to 2 more)
**Any serious adverse event**											
21977 (1 RCT)	serious[b,c]	not serious	not serious	serious[d]	none	⊕⊕◯◯ Low	12/5476 (0.2%)	43/16501 (0.3%)	**RR 1.19** (0.63 to 2.25)	2 per 1,000	**0 fewer per 1,000** (from 1 fewer to 3 more)
**Related unsolicited serious adverse event**											
21977 (1 RCT)	serious[b,c]	not serious	not serious	serious[d]	none	⊕⊕◯◯ Low	1/5476 (0.0%)	0/16501 (0.0%)	**RR 0.11** (0.00 to 2.72)	0 per 1,000	**0 fewer per 1,000** (from 0 fewer to --)

CI: confidence interval; **RR:** risk ratio

Explanations

a. The primary and secondary outcomes were reported following the per-protocol analysis approach.
b. Study reported serious adverse events but did not report full adverse events data.
c. Safety outcomes were not reported for all participants who received at least one dose.

d. The 95% confidence intervals for this outcome were wide.
e. The 95% confidence interval for this outcome includes the possibility of no effect.

Supplemental Table S2

Certainty assessment							Summary of findings				
Participants (studies) Follow-up	Risk of bias	Inconsistency	Indirectness	Imprecision	Publication bias	Overall certainty of evidence	Study event rates (%)		Relative effect (95% CI)	Anticipated absolute effects	
							With placebo	With Astrazeneca		Risk with placebo	Risk difference with Astrazeneca
**Any symptomatic cases**											
23848 (1 RCT)	very serious[a,b]	not serious	not serious	not serious	none	⨁⨁◯◯ Low	150/11766 (1.3%)	63/12082 (0.5%)	**RR 0.41** (0.31 to 0.55)	13 per 1,000	**8 fewer per 1,000** (from 9 fewer to 6 fewer)
**Symptomatic cases after effective period**											
23848 (1 RCT)	very serious[a,b]	not serious	not serious	not serious	none	⨁⨁◯◯ Low	112/11766 (1.0%)	37/12082 (0.3%)	**RR 0.32** (0.22 to 0.47)	10 per 1,000	**6 fewer per 1,000** (from 7 fewer to 5 fewer)
**Severe cases after effective period**											
23848 (1 RCT)	very serious[a,b]	not serious	not serious	serious[c,d]	none	⨁◯◯◯ Very low	1/11766 (0.0%)	0/12082 (0.0%)	**RR 0.32** (0.01 to 7.97)	0 per 1,000	**0 fewer per 1,000** (from 0 fewer to 1 more)

Supplemental Table S2

Certainty assessment							Summary of findings			
**Covid deaths**										
23848 (1 RCT)	serious[b]	not serious	not serious	serious[c,d]	none	⊕⊕◯◯ Low	1/11766 (0.0%)	0/12082 (0.0%)	**RR 0.33** (0.01 to 7.98)	0 per 1,000	**0 fewer per 1,000** (from 0 fewer to 1 more)
**All-cause mortality**										
23848 (1 RCT)	serious[b]	not serious	not serious	serious[c,d]	none	⊕⊕◯◯ Low	4/11766 (0.0%)	1/12082 (0.0%)	**RR 0.24** (0.03 to 2.18)	0 per 1,000	**0 fewer per 1,000** (from 0 fewer to 0 fewer)
**Any serious adverse event**										
23745 (1 RCT)	serious[b]	not serious	not serious	serious[c]	none	⊕⊕◯◯ Low	78/11724 (0.7%)	77/12021 (0.6%)	**RR 0.96** (0.70 to 1.32)	7 per 1,000	**0 fewer per 1,000** (from 2 fewer to 2 more)
**Related unsolicited serious adverse event**										
23745 (1 RCT)	serious[b]	not serious	not serious	serious[c]	none	⊕⊕◯◯ Low	1/11724 (0.0%)	1/12021 (0.0%)	**RR 0.98** (0.06 to 15.59)	0 per 1,000	**0 fewer per 1,000** (from 0 fewer to 1 more)

CI: confidence interval; **RR:** risk ratio

Explanations

a. Data for this outcome were not available for all participants randomized.
b. Three of four included trials were single blind studies.
c. The 95% confidence intervals for this outcome were wide.
d. The 95% confidence interval for this outcome includes the possibility of no effect.

Legend

Supplemental Table S1. GRADE evidence profile denoting the benefits and risks associated with Sputnik vaccine for the prevention of COVID-19 in adults. The table includes relative and absolute effects along with the overall certainty of the evidence.

Supplemental Table S2. GRADE evidence profile denoting the benefits and risks associated with Astrazeneca vaccine for the prevention of COVID-19 in adults. The table includes relative and absolute effects along with the overall certainty of the evidence.
